# Comparative Analysis of Pedicle Screw Fixation and Interspinous Devices in Lumbar Spinal Fusion: Clinical and Surgical Outcomes in Degenerative Spine Conditions

**DOI:** 10.3390/jpm15030095

**Published:** 2025-02-28

**Authors:** Vittorio Orlando, Gianluca Galieri, Edoardo Mazzucchi, Fabrizio Pignotti, Antonella Carcagnì, Paola Bazzu, Roberto Altieri, Manlio Barbarisi, Alessandro Olivi, Giovanni Sabatino, Giuseppe La Rocca

**Affiliations:** 1 Institute of Neurosurgery, Fondazione Policlinico Universitario A. Gemelli IRCCS, Catholic University, 00168 Rome, Italy; vittorio.orlando01@icatt.it (V.O.); alessandro.olivi@policlinicogemelli.it (A.O.); giovanni.sabatino@policlinicogemelli.it (G.S.); giuseppe.larocca@policlinicogemelli.it (G.L.R.); 2 Neurosurgical Training Center and Brain Research, Mater Olbia Hospital, 07026 Olbia, Italy; edoardo.mazzucchi@ifo.it (E.M.); fabrizio.pignotti@materolbia.com (F.P.); 3 Department of Neurosurgery, IRCCS Regina Elena National Cancer Institute, 00144 Rome, Italy; 4 Department of Neurosurgery, Mater Olbia Hospital, 07026 Olbia, Italy; 5 Facility of Epidemiology and Biostatistics–Gemelli Generator, Fondazione Policlinico Universitario A. Gemelli IRCCS, 00168 Rome, Italy; antonella.carcagni@policlinicogemelli.it; 6 Clinical Psychology Service, Mater Olbia Hospital, 07026 Olbia, Italy; paola.bazzu@materolbia.com; 7 Multidisciplinary Department of Medical-Surgical and Dental Specialties, University of Campania “Luigi Vanvitelli”, 81100 Caserta, Italy; roberto.altieri@unicampania.it (R.A.); manlio.barbarisi@unicampania.it (M.B.)

**Keywords:** interspinous stabilization, pedicle screw fusion, degenerative lumbar instability, minimally invasive spine surgery, psychological assessment

## Abstract

**Background/Objectives**: Degenerative lumbar spine conditions are a major cause of disability, particularly in elderly patients or those with comorbidities. Surgical treatment often combines decompression and stabilization to address pain and instability. Pedicle screws are the gold standard for stabilization but pose challenges in patients with compromised bone quality. Interspinous devices have emerged as a less invasive alternative, but comparative studies are limited. This study aimed to compare clinical and surgical outcomes of lumbar decompression with stabilization using pedicle screws versus interspinous devices. **Methods**: A retrospective cohort study was conducted on patients who underwent lumbar decompression with either pedicle screw fixation or interspinous device stabilization at Mater Olbia Hospital between February 2020 and February 2023. Outcomes were evaluated using VAS for back and leg pain, SF-36 for quality of life, EQ-5D, and SCL-90 for psychological factors. Statistical analysis included paired *t*-tests, chi-square tests, and multivariate regression. **Results**: A total of 728 patients were included. The interspinous device group consisted of older patients with higher comorbidity burdens (mean age: 68.4 vs. 59.2 years, *p* < 0.001). Surgical time and incision size were significantly shorter in the interspinous group (*p* < 0.001), and no postoperative complications were reported, compared to 3.5% in the pedicle screws group (*p* < 0.05). Both groups demonstrated significant improvements in pain (VAS), quality of life (SF-36, EQ-5D), and psychological outcomes (SCL-90). Somatization and paranoid ideation were significant predictors of worse postoperative pain, particularly in the pedicle screws group. No significant differences in quality-of-life improvements were observed between the groups. **Conclusions**: Both stabilization techniques are effective for lumbar spine surgery, with interspinous devices offering a safer and less invasive option for high-risk patients. Psychological factors significantly influence pain outcomes, underscoring the need for a comprehensive approach addressing both physical and psychological aspects to optimize patient recovery.

## 1. Introduction

Degenerative spine conditions represent a significant cause of disability and reduced quality of life, particularly in elderly patients or those with comorbidities such as osteoporosis [1,2]. These conditions often involve a combination of chronic pain, neurological deficits, and functional impairment, necessitating timely and effective management [3,4,5,6]. The lumbar spine is especially prone to degenerative changes due to its biomechanical load-bearing role, making it a frequent site of surgical intervention [7,8]. Historically, surgical approaches have focused on decompressing the neural elements to alleviate pain and neurological symptoms, often complemented by stabilization techniques to address spinal instability [9,10,11].

Lumbar spine instability, characterized by excessive motion between vertebral segments, plays a central role in the progression of degenerative conditions [12,13]. Traditional stabilization techniques, such as pedicle screw and rod systems, have been widely regarded as the gold standard for achieving biomechanical stability after decompression [14]. These systems provide robust fixation, facilitating spinal fusion and long-term biomechanical integrity [15]. However, their application is not without challenges, particularly in elderly patients with osteoporosis or other factors compromising bone quality. In such cases, the risk of hardware failure, pseudoarthrosis, and surgical complications may be elevated, prompting the exploration of alternative stabilization methods [16].

Interspinous devices have emerged as a promising alternative in select patient populations. Initially developed as simple extension-blocking devices to offload affected spinal segments, these systems have evolved into sophisticated interspinous fixation devices (IFDs) [16]. Modern designs provide both decompression and stabilization while minimizing invasiveness [17]. By distributing biomechanical stress across treated spinal segments and preserving adjacent segment mobility, interspinous devices aim to reduce the morbidity associated with traditional stabilization techniques [18].

The rationale for utilizing interspinous devices lies in their potential to offer effective stabilization with a less invasive surgical footprint. This is particularly advantageous for patients at higher surgical risk due to advanced age, osteoporosis, or multiple comorbidities. Despite their increasing adoption, the comparative efficacy of interspinous devices versus traditional pedicle screw systems in achieving clinical and surgical outcomes remains a subject of ongoing investigation [19].

This study aims to evaluate and compare the clinical and surgical outcomes of lumbar fusion using pedicle screws and rods with those achieved using the interspinous device in selected populations, both performed in conjunction with spinal decompression. By examining these two approaches, the study seeks to provide evidence regarding the safety, effectiveness, and appropriate indications for interspinous devices, ultimately contributing to more personalized treatment strategies for patients with degenerative lumbar spine conditions.

## 2. Materials and Methods

### 2.1. Study Design

This is a retrospective cohort study conducted at the Mater Olbia Hospital, Department of Neurosurgery. The study compares two groups of patients who underwent lumbar spinal decompression with subsequent stabilization using either pedicle screws and rods or an interspinous device. Ethical approval was obtained from the Ethics Committee of the Autonomous Region of Sardinia (protocol number 276/2020/CE of 29 October 2020). The study period spanned from October 2020 to February 2023. Patients were followed for at least one year postoperatively to monitor outcomes.

### 2.2. Data Collection

Data were collected from electronic medical records and included demographic information, clinical history, operative details, and postoperative outcomes. Parameters analyzed included age, sex, body mass index (BMI), comorbidities, duration of hospital stay, surgical time, and intraoperative and postoperative complications. Clinical outcomes were assessed using validated instruments such as the Visual Analog Scale (VAS) for back pain and leg pain [20], the Oswestry Disability Index (ODI) [21], the Short Form-36 (SF-36) [22] and EuroQuality of life- 5 Dimensions (EQ-5D). Questionnaires were administered preoperatively, at one month postoperatively, and at the one-year follow-up. Beyond this period, patients were followed clinically.

### 2.3. Inclusion and Exclusion Criteria

Inclusion criteria:

- Patients aged 18 years and older.
- Diagnosed with lumbar spinal stenosis with instability requiring surgical intervention. Instability comprised facet joint effusion, intervertebral disc degeneration, laxity of the posterior longitudinal ligament, or severe atrophy of the paravertebral musculature, as well as Meyerding Grade I listhesis observed on dynamic lumbar spine X-rays.
- Underwent lumbar decompression with either pedicle screw fixation or interspinous device stabilization.
- Availability of complete preoperative and postoperative clinical data.
- Follow-up at least one year.

Exclusion criteria:

- Prior lumbar spine surgery at the affected level.
- Severe systemic comorbidities precluding any surgical intervention.
- Incomplete follow-up data.
- Traumatic lumbar spine conditions.
- Oncologic vertebral pathologies.
- Infectious vertebral conditions (e.g., discitis, spondylodiscitis).

Eligibility for the interspinous device group:

- Patients over 70 years with significant comorbidities.
- Patients over 50 years diagnosed with osteoporosis (confirmed by Dual- Energy X-ray Absorptiometry [DXA] scan and T-score < −2.5).
- Patients unsuitable for long surgical procedures (≤1 h of surgical time due to high anesthetic risk).

### 2.4. Surgical Treatments

All surgeries were conducted by a consistent team of two experienced surgeons to ensure uniformity in the surgical approach and technique.

Group 1: Patients underwent percutaneous stabilization using pedicle screws and rods followed by lumbar decompression. This technique involved bilateral pedicle screw placement, rod fixation, and bone grafting to promote fusion. The surgical procedure adhered to the method described in the study by La Rocca et al. [23].

Group 2: Patients underwent lumbar decompression followed by stabilization using the Aspen^®^ interspinous device. The device was positioned between the spinous processes of the affected levels, providing segmental stability while preserving adjacent segment mobility. The surgical technique followed the protocol outlined by La Rocca et al. [24].

The Aspen^®^ device (Zimmer Biomet Spine, Inc., Westminster, CO, USA) is a titanium interspinous fixation system designed to offer segmental stability following decompression. Its unique design features an adjustable interlocking mechanism, enabling secure fixation while minimizing biomechanical stress on the treated spinal segment. The device is inserted through a minimally invasive approach, reducing tissue disruption and preserving the integrity of adjacent spinal structures [25].

### 2.5. Statistical Analysis

Data were analyzed using R software (version 4.4.2) [26]. Continuous variables were presented as mean ± standard deviation (SD) or median (interquartile range [IQR]) based on data distribution [27]. The normality of distributions was assessed using the Shapiro–Wilk test. Comparisons between groups were conducted using Student’s *t*-test for normally distributed continuous variables and the Mann–Whitney U test for non-normally distributed variables [28]. Categorical variables were expressed as frequencies and percentages and analyzed using the chi-square test or Fisher’s exact test as appropriate.

Multivariate analysis was performed to adjust for potential confounding factors, with regression linear models employed to evaluate predictors of clinical outcomes [29]. Pre- and postoperative differences within groups were assessed using paired *t*-tests for normally distributed data or Wilcoxon signed-rank tests for non-parametric data. Results were considered statistically significant at a *p*-value < 0.05. Effect sizes (e.g., Cohen’s d for continuous variables) were calculated to provide a measure of clinical relevance where applicable [30].

## 3. Results

### 3.1. Demographic and Clinical Characteristics

The demographic and clinical characteristics of the study population are summarized in Table 1. A total of 728 out of 756 patients completed the follow-up and were included in the study, with 356 patients in the interspinous device stabilization group and 372 in the pedicle screws stabilization group. The interspinous device group included older patients (mean age: 68.4 ± 9.04 years) compared to the pedicle screws group (mean age: 59.2 ± 10.2 years; *p* < 0.001). BMI was slightly higher in the pedicle screws group (27.4 ± 4.64) compared to the interspinous device group (26.5 ± 4.44; *p* < 0.01). No significant differences were observed in gender distribution or comorbidities between groups. The mean follow-up duration was 41.2 ± 10.6 months.

### 3.2. Surgical and Postoperative Outcomes

The surgical characteristics and hospitalization data are summarized in Table 2. The surgical time was significantly shorter in the interspinous device group (mean: 39 ± 12 min) compared to the pedicle screws group (mean: 72 ± 20 min; *p* < 0.001). Similarly, the incision size was smaller in the interspinous device group (mean: 4.56 ± 1.69 cm) than in the pedicle screws group (mean: 8.91 ± 1.70 cm; *p* < 0.001). The rate of intraoperative complications was comparable between groups (1.12% vs. 1.34%; *p* > 0.05); however, postoperative complications were slightly higher in the pedicle screws group (1.88% vs. 0.56%; *p* < 0.05).

In the interspinous device group, the four intraoperative complications included two cases of Cerebrospinal Fluid (CSF) leak and two spinous process fractures. In the pedicle screws group, the five intraoperative complications consisted of three cases of CSF leak and two cases of screw repositioning. Regarding postoperative complications, the two cases in the interspinous device group were both subfascial hematomas, while in the pedicle screws group, the seven postoperative complications included two subfascial hematomas, three cases of postoperative anemia, and two infections managed conservatively with antibiotic therapy.

### 3.3. Functional and Quality of Life Outcomes

VAS scores for back and leg pain significantly improved postoperatively and at follow up in both groups with *p* value < 0.001 for all comparisons (Table 3). The mean reduction in VAS leg pain was 6.1 ± 2.1 in the interspinous group and 6.07 ± 2.0 in the pedicle screws group, with no statistically significant difference between the groups (*p* = 0.87). Similarly, both groups showed marked improvements in back pain (mean reductions: interspinous 5.14 vs. pedicle screws 5.01; *p* = 0.526) (Table 4).

Regarding the SCL-90 scales, statistically significant difference between groups were observed for anxiety (*p* = 0.048), depression (*p* = 0.017), psychoticism (*p* = 0.010) and the Global Severity Index (GSI) (*p* = 0.011). In addition, improvements in SF-36 scores were significant across all domains in both groups (*p* < 0.0001) (Table 3).

Social functioning, pain and general health scores were slightly higher in the interspinous group at follow-up, though when comparing preoperative and follow-up differences between the groups, no significant differences emerged (Table 4).

The Euro Quality of life 5-Dimension (EQ-5D) index also improved significantly postoperatively and at follow-up in both groups (*p* < 0.0001), with no significant difference in the extent of improvement between the interspinous group (−0.31) and the pedicle screws group (−0.29; *p* = 0.253; Table 4).

The multivariate regression analysis revealed important associations between postoperative outcomes—VAS back pain, VAS leg pain, and EQ-5D—and clinical, psychological, and demographic variables in the interspinous and pedicle screws groups (Supplementary Materials—Tables S1–S3).

For VAS leg pain, somatization (*p* = 0.001) emerged as a significant predictor of worse outcomes in the overall cohort and particularly in the pedicle screws group. Paranoid ideation (*p* = 0.014) and Psychoticism (*p* = 0.046) also significantly influenced outcomes in the pedicle screws group, highlighting the impact of psychological factors on postoperative pain. Furthermore, improvements in physical functioning and pain domains of the SF-36 were correlated with reductions in leg pain for both groups, underscoring the contribution of physical recovery to pain relief. However, no significant differences between the interspinous and pedicle screws groups were observed when these SF-36 domains were directly compared.

Similarly, for VAS back pain, somatization (*p* = 0.020) was associated with worse outcomes in both the overall cohort and the pedicle screws group. Additional psychological factors, such as phobic anxiety (*p* = 0.007) and paranoid ideation (*p* = 0.006), significantly influenced back pain outcomes in the pedicle screws group. Physical recovery also played a pivotal role, with improvements in the SF-36 domains of role limitations due to physical health (*p* < 0.0001) and mental health (*p* = 0.010) being strongly associated with reductions in back pain. These findings suggest an interplay between physical and psychological factors in determining postoperative pain relief.

For EQ-5D, no significant associations were found with psychological variables. Instead, physical health, as measured by the SF-36, emerged as a primary determinant of quality-of-life improvements in both groups. Statistically significant relationship between EQ-5D and Role limitation due to physical health (*p* = 0.021), emotional problems (*p* = 0.012), energie fatigue (*p* = 0.013), emotional well-being (*p* = 0.013), social functioning pain, general (*p* = 0.010) physical (*p* = 0.003) and mental (*p* = 0.013) health were showed in interspinous group. Interestingly, BMI showed a borderline significant negative association with EQ-5D in the overall cohort and in the interspinous group, suggesting that higher BMI may slightly limit perceived quality-of-life improvements.

Regarding demographic factors, age did not consistently predict postoperative outcomes across groups. However, a borderline trend was noted in the interspinous group, where increased age appeared to slightly reduce the improvement in VAS leg pain. BMI, on the other hand, did not significantly influence VAS back pain or leg pain outcomes but showed a weak negative association with physical health recovery in the interspinous group and with quality-of-life improvements in the overall cohort.

## 4. Discussion

Recognizing the broad spectrum of complications associated with the evolution of pedicle screws, it’s evident that not all patients are suited for such stabilization techniques [31]. When inserted via the classically minimally invasive midline posterior techniques, interspinous fixation devices (IFDs) provide good resistance to flexion and extension and moderate resistance to lateral bending and axial rotation; thus, these devices are well-suited for spinal fusion and stabilization [32]. A randomized, controlled, multi-center clinical trial has shown that the Aspen^®^ System could be a significantly faster and less invasive alternative to pedicle screw fixation in support of interbody fusion [33]. Moreover, it has been demonstrated in biomechanical studies that the implant does not significantly alter the kinematics of the motion segments adjacent to the instrumented level [34].

Interspinous devices have been combined with procedures such as microdiscectomy, foraminotomy, and interbody fusion. Their use has also been suggested in cases involving unilateral or total facetectomy to provide stabilization. However, there are a lack of biomechanical studies confirming the stability achieved by interspinous devices after unilateral facetectomy, which is known to have a destabilizing effects on the spine [35]. Gonzalez-Blohm et al. analyzed the biomechanical properties of interspinous fusion devices used as standalone solutions, adjuncts to lumbar decompression surgeries, and supplemental support in posterior lumbar interbody fusion (PLIF) constructs. Their findings indicate that IFDs effectively restore flexion-extension stability after unilateral laminotomies. While PLIF constructs combined with IFDs and pedicle screws demonstrated comparable stability in flexion-extension and axial rotation, bilateral pedicle screw PLIF constructs showed superior resistance to lateral bending forces [36]. This study reinforces the growing body of evidence suggesting that interspinous devices, such as the Aspen^®^, offer distinct advantages over traditional pedicle screw systems for specific patient populations. The results of this study, in line with previous findings from Gazzeri et al., demonstrate that interspinous devices provide effective stabilization while maintaining a less invasive surgical profile [37].

One notable advantage of the interspinous fusion device is its significant reduction in operative time and intraoperative blood loss compared to pedicle screw fixation. The shorter duration of surgery and minimally invasive nature of the procedure are particularly beneficial for elderly patients or those with significant comorbidities. These findings underscore the importance of tailoring surgical approaches to patient-specific needs, particularly in high-risk populations such as the elderly.

In terms of clinical outcomes, patients treated with the interspinous fusion device exhibited superior improvements in back pain compared to those treated with pedicle screws. This aligns with the biomechanical advantages of interspinous devices, which are designed to unload stress on the posterior annulus and reduce intradiscal pressure. Additionally, the preservation of surrounding anatomical structures and avoidance of extensive paraspinal muscle dissection contribute to improved postoperative recovery and reduced complications.

Despite these advantages, the use of interspinous devices is not without limitations. Concerns regarding adjacent segment degeneration (ASD) have been raised in the literature. While the reduced rigidity of interspinous devices may protect against hardware-related complications, it could predispose patients to ASD over the long term. Gazzeri et al. reported a higher incidence of ASD in patients treated with interspinous devices compared to those with pedicle screws [38]. Further long-term studies are needed to better understand the implications of this finding and optimize patient selection criteria.

Although interspinous devices have demonstrated effectiveness in early outcomes, concerns about long-term complications, such as sinking into or erosion of the spinous process, have been reported. In our experience, follow-up radiological imaging showed gradual and complete ossification of the interspinous device, with no significant low back pain reported by patients. To mitigate these risks, future studies should focus on improving device design to minimize stress on the spinous processes and evaluating strategies to enhance the long-term durability of interspinous devices.

Moreover, the psychological and functional outcomes observed in this study, assessed using the SCL-90 and SF-36 scales, highlight the holistic benefits of the Aspen^®^ device. Improvements in anxiety, depression, and overall quality of life were observed in both groups, with no statistically significant differences between them. These results emphasize that while the choice of surgical technique is crucial, patient-centered care that addresses psychological and functional recovery is equally important [39,40].

The interspinous device group included older patients with higher comorbidity burdens compared to the pedicle screws group. This aligns with previous studies, which have recommended interspinous devices for patients with increased surgical risk or reduced bone quality (e.g., osteoporosis) [41]. The significant age difference underscores the utility of interspinous devices as a less invasive alternative for patients unable to tolerate longer or more complex procedures. However, the benefits of reduced surgical invasiveness must be balanced against the potential for less robust stabilization compared to pedicle screws in certain patient populations [42].

Both groups demonstrated significant postoperative and follow-up improvements in pain and quality of life, with no major differences in the extent of improvement. This supports the growing body of evidence that both stabilization techniques are effective in appropriately selected patients. Notably, the significantly lower rate of postoperative complications in the interspinous group (0.56%) compared to the pedicle screws group (1.88%; *p* < 0.05) underscores the safety profile of this approach, particularly in elderly or frail patients.

## 5. Limitations and Future Directions

Despite the strengths of this study, including its robust statistical analysis and detailed patient stratification, several limitations should be acknowledged. First, the retrospective design may introduce selection bias. Second, the follow-up period, while sufficient for early outcomes, does not capture long-term durability of the stabilization techniques. Third, patients selected for interspinous devices were generally older and had more comorbidities, making them less suitable for traditional pedicle screw fixation. This intentional selection of higher-risk patients, while clinically justified, may limit the generalizability of the findings. Interspinous devices were preferentially used in patients with advanced age (>70 years), osteoporosis (T-score < −2.5), or high anesthetic risks, reflecting a less invasive alternative. Consequently, the observed outcomes for interspinous devices may underestimate their potential benefits in a broader patient population. Future prospective, randomized studies are needed to validate these findings and explore the impact of these surgical approaches on adjacent segment degeneration and long-term patient satisfaction.

## 6. Conclusions

In conclusion, this study demonstrates that both pedicle screws and interspinous devices are viable options for lumbar spinal stabilization, with distinct advantages depending on patient characteristics and surgical goals. The choice of technique should be individualized, considering patient age, comorbidities, and anatomical factors. Interspinous devices offer a valuable alternative for patients at higher surgical risk, while pedicle screws remain the gold standard for robust stabilization in younger, healthier patients. The analysis underscores the significant impact of psychological factors on postoperative pain outcomes, highlighting the importance of adopting a comprehensive approach that integrates both physical and psychological aspects to enhance surgical outcomes in patients undergoing lumbar stabilization.

## Figures and Tables

**Table 1:** Demographic and clinical variables. SD (standard deviation); BMI (body mass index).

Variable	Overall (*n* = 728)	Interspinous (*n* = 356)	Pedicle Screws (*n* = 372)
Gender (Male, %)	610 (83.9%)	296 (83.1%)	314 (84.6%)
Age (years, mean ± SD)	63.7 ± 10.7	68.4 ± 9.04	59.2 ± 10.2
Weight (kg)	75.2 ± 15.5	74.4 ± 15.6	76.0 ± 15.3
Height (m)	1.67 ± 0.08	1.67 ± 0.08	1.66 ± 0.08
BMI (kg/m^2^)	26.9 ± 4.6	26.5 ± 4.44	27.4 ± 4.64
Hypertension (%)	345 (47.4%)	198 (55.6%)	147 (39.5%)
Diabetes Mellitus II (%)	98 (13.5%)	43 (12.1%)	55 (14.8%)
Cardiovascular Disease (%)	234 (32.1%)	135 (37.9%)	99 (26.6%)
Osteoarthritis (%)	150 (20.6%)	74 (20.8%)	76 (20.4%)
Previous Spinal Surgery (%)	107 (14.7%)	46 (12.9%)	61 (16.4%)
Autoimmune Diseases (%)	98 (13.5%)	42 (11.8%)	56 (15.1%)
Neoplasms (%)	73 (10%)	35 (9.8%)	38 (10.2%)
Smokers (%)	239 (32.8%)	111 (31.2%)	128 (34.4%)

**Table 2:** Hospitalization and surgery.

Variable	Overall (*n* = 728)	Interspinous (*n* = 356)	Pedicle Screws (*n* = 372)
Pathology (%)			
- Stenosis with instability	728 (100%)	356 (48.9%)	372 (51.1%)
Operated Level (%)			
- L2–L3	16 (2.2%)	15 (4.2%)	1 (0.3%)
- L3–L4	90 (12.4%)	57 (16.0%)	33 (8.9%)
- L4–L5	528 (72.5%)	244 (68.5%)	284 (76.3%)
- L5–S1	93 (12.8%)	40 (11.2%)	53 (14.2%)
Hospital Stay (days)	2.7 ± 0.81	2.7 ± 0.81	2.6 ± 0.70
Preoperative Analgesia (%)	32 (4.4%)	0	32 (8.6%)
Intraoperative Complications (%)	9 (1.24%)	4 (1.12%)	5 (1.34%)
Postoperative Complications (%)	9 (1.24%)	2 (0.56%)	7 (1.88%)
Surgical time (minute)	55.9 ± 24.5	39 ± 12	72 ± 20
Wound Size (cm)	6.78 ± 2.76	4.56 ± 1.69	8.91 ± 1.70
Drain Usage (%)	390 (53.7%)	27 (7.6%)	363 (97.8%)

**Table 3:** Comparative analysis of preoperative, postoperative and follow-up outcomes for interspinous devices and pedicle screws. EQ-5D (Euro Quality of life 5 Dimension); SCL-90 (Symptom Checklist-90), GSI (Global Severity Index); PST (Positive Symptom Total); PSDI (Positive Symptom Distress Index); CSI (Current Symptom Index); SF-36 (Short Form 36); FU (Follow-up); SD (Standard Deviation).

	Interspinous				Pedicle Screws			
	Baseline (Mean ± SD)	Post-Operative (Mean ± SD	FU (Mean ± SD)	*p*-Value	Baseline (Mean ± SD)	Post-Operative (Mean ± SD	FU (Mean ± SD)	*p*-Value
VAS								
Leg Pain	8.11 (1.37)	2.19 (1.9)	2.01 (2.1)	<0.0001	8.06 (1.57)	2.15 (2.46)	1.99 (2.00)	<0.0001
Back Pain	8.10 (1.37)	2.98 (2.7)	2.96 (2.47)	<0.0001	7.91 (1.61)	3.67 (2.1)	2.90 (2.25)	<0.0001
EQ-5D	0.37 (0.21)	0.54 (0.25)	0.69 (0.23)	<0.0001	0.38 (0.21)	0.57(0.20)	0.68 (0.21)	<0.0001
SCL90								
Depression	0.8 (0.6)				0.6 (0.5)			0.017
Anxiety	0.6 (0.61)				0.48 (0.5)			0.048
Psychoticism	0.33 (0.41)				0.25 (0.4)			0.010
GSI	1.76 (6.52)				0.81 (3.0)			0.011
PST	30.6 (18.3)				28.9 (17.5)			0.329
PSDI	1.83 (0.70)				1.77 (0.64)			0.219
CSI	0.65 (0.50)				0.59 (0.44)			0.054
SF-36								
Physical Functioning	30.3 (24)		52.3 (25.5)	<0.0001	33.5 (25.1)		57.9 (27.7)	<0.0001
Pain	20.8 (17.8)		48.09 (26.4)	<0.0001	19.7 (18.9)		45.3 (24.8)	<0.0001
General Health	46.1 (18.2)		59.3 (16.2)	<0.0001	50.3 (19.1)		62.0 (17)	<0.0001
Physical Health	32.9 (17)		50.1 (18.5)	<0.0001	35.8 (17.6)		53.1 (20.1)	<0.0001
Mental Health	47.8 (17)		60.5 (17.9)	<0.0001	50.4 (20)		62.9 (19.7)	<0.0001

**Table 4:** T-Student Test for comparation of preoperative and follow-up differences between interspinous device and pedicle screws. VAS (Visual Analog Scale); EQ-5D (Euro Quality of life 5 Dimension); SF-36 (Short Form 36).

	Interspinous Mean Pre- and Follow-Up Value	Pedicle Screws Mean Pre- and Follow-Up Value	*p*-Values
VAS			
Leg pain	6.10	6.07	0.873
Back pain	5.14	5.01	0.526
EQ-5D	−0.31	−0.29	0.253
SF-36			
Physical Functioning	22.08	24.41	0.300
Role limitation due to physical health	6.61	3.49	0.339
Role limitation due to emotional problems	12.76	17.56	0.258
Energie fatigue	13.52	13.86	0.848
Emotional well being	9.45	10.11	0.678
Social Functioning	16.71	12.93	0.101
Pain	27.19	25.56	0.472
General Health	13.17	11.73	0.306
Physical Health	17.29	17.26	0.987
Mental Health	12.64	12.59	0.970

## Data Availability

The data presented in this study are available on request from the corresponding author.

## Supplementary Materials

The following supporting information can be downloaded at: https://www.mdpi.com/article/10.3390/jpm15030095/s1; Table S1: Multivariate Regression Analysis: Clinical and SCL-90 Factors for VAS Leg Pain and SF-36 Outcomes, Comparing Interspinous Devices and Pedicle Screw Stabilizations.; Table S2: Multivariate Regression Analysis: Clinical and SCL-90 Factors for VAS Back Pain and SF-36 Outcomes, Comparing Interspinous Devices and Pedicle Screw Stabilizations; Table S3: Multivariate Regression Analysis: Clinical and SCL-90 Factors for EQ-5D and SF-36 Outcomes, Comparing Interspinous Devices and Pedicle Screw Stabilizations.

## Abbreviations

The following abbreviations are used in this manuscript:

BMI	Body Mass Index
CSF	Cerebrospinal Fluid
CSI	Current Symptom Index
EQ-5D	EuroQol-5 Dimensions
GSI	Global Severity Index
IQR	Interquartile Range
ODI	Oswestry Disability Index
PSDI	Positive Symptom Distress Index
PST	Positive Symptom Total
SCL-90	Symptom Checklist 90
SD	Standard Deviation
SF-36	Short Form 36
VAS	Visual Analog Scale
VAS-BP	Visual Analog Scale for Back Pain
VAS-LP	Visual Analog Scale for Leg Pain
IFDs	Interspinous Fixation Devices
PLIF	Posterior Lumbar Interbody Fusion
ASD	Adjacent Segment Degeneration
DXA	Dual-energy X-ray Absorptiometry
