# Supplementary material for: Comparative Analysis of Pedicle Screw Fixation and Interspinous Devices in Lumbar Spinal Fusion: Clinical and Surgical Outcomes in Degenerative Spine Conditions
Source: J Pers Med. 2025 Feb 28;15(3):95. doi: 10.3390/jpm15030095 (PMC11943498; doi:10.3390/jpm15030095)
Supplement: Supplementary file 1 [file jpm-15-00095-s001.zip › jpm-3450716-supplementary.pdf]

Supplementary Materials

Table S1. Multivariate Regression Analysis: Clinical and SCL-90 Factors for VAS Leg Pain and SF-36 Outcomes, Comparing Interspinous Devices and Pedicle Screw Stabilizations. BMI (Body Mass Index); DMII (Diabetes Mellitus II), SCL-90 (Symptom Checklist-90), GSI (Global Severity Index); PST (Positive Symptom Total); PSDI (Positive Symptom Distress Index); CSI (Current Symptom Index); SF-36 (Short Form 36); SD (Standard Deviation).

Vas leg pain	Overall	P-values	Interspinous device	P-values	Screws and Rods	P-values
			Estimate (sd)		Estimate(sd)	
Age	0.010 (0.005)	0.050	0.015 (0.008)	0.067	0.013 (0.008)	0.112
BMI	-0.002 (0.012)	0.851	-0.002 (0.017)	0.865	-0.006 (0.017)	0.701
DM.II	-0.037 (0.164)	0.818	0.153 (0.231)	0.509	-0.132 (0.238)	0.579
Fibromyalgia	0.206 (0.374)	0.582	-0.053 (0.533)	0.921	0.383 (0.532)	0.472
Others orthopedic diseases	0.044 (0.137)	0.743	0.152 (0.185)	0.411	-0.034 (0.207)	0.867
**SCL90**						
Somatization	0.619 (0.194)	0.001	0.288 (0.255)	0.258	0.985 (0.298)	**0.001**
Obsessiveness	0.120 (0.210)	0.569	-0.133 (0.291)	0.648	0.431 (0.311)	0.167
Sensitivity	0.244 (0.221)	0.269	0.144 (0.289)	0.618	0.331 (0.342)	0.334
Depression	0.032 (0.227)	0.887	-0.046 (0.299)	0.877	0.158 (0.351)	0.651
Hostility	-0.081 (0.201)	0.684	-0.315 (0.282)	0.264	0.080 (0.290)	0.782
Fobic Anxiety	0.070 (0.246)	0.774	-0.424 (0.315)	0.180	0.593 (0.387)	0.127
Paranoid Ideation	0.199 (0.161)	0.215	-0.137 (0.203)	0.500	0.643 (0.261)	**0.014**
Psychoticism	-0.467 (0.284)	0.100	-0.049 (0.378)	0.895	-0.879 (0.440)	**0.046**
GSI	-0.003 (0.012)	0.765	-0.004 (0.013)	0.749	0.013 (0.039)	0.735
PST	-0.008 (0.008)	0.324	0.0001 (0.010)	0.992	-0.017 (0.012)	0.177
PSDI	-0.070 (0.113)	0.533	-0.043 (0.148)	0.767	-0.059 (0.174)	0.733
CSI	-0.215 (0.717)	0.764	0.764 (0.918)	0.406	-1.387 (1.150)	0.228
**SF-36**						
Age	0.005 (0.004)	0.268	0.006 (0.007)	0.374	0.007 (0.007)	0.365
BMI	-0.010 (0.010)	0.330	-0.021 (0.014)	0.147	-0.004 (0.016)	0.788
DM.II	-0.044 (0.145)	0.761	0.146 (0.196)	0.458	-0.213 (0.220)	0.332
Fibromyalgia	0.361 (0.335)	0.281	0.313 (0.460)	0.496	0.393 (0.490)	0.423
Others orthopedic diseases	0.126 (0.120)	0.294	0.241 (0.156)	0.123	-0.006 (0.188)	0.972
Role limitation due to physical health	0.004 (0.002)	**0.048**	0.002 (0.003)	0.564	0.007 (0.003)	**0.038**
Role limitation due to emotional problems	0.082 (0.073)	0.261	0.047 (0.090)	0.600	0.147 (0.119)	0.217
Energie fatigue	0.111 (0.097)	0.255	0.066 (0.119)	0.577	0.192 (0.158)	0.225
Emotional well being	0.128 (0.121)	0.293	0.067 (0.151)	0.655	0.237 (0.198)	0.230
Social Functioning	0.053 (0.048)	0.276	0.032 (0.060)	0.587	0.092 (0.079)	0.242

Pain	-0.026 (0.003)	**0.0001**	-0.029 (0.004)	**0.0001**	-0.022 (0.005)	**0.0001**
General Health	0.130 (0.121)	0.282	0.072 (0.150)	0.631	0.240 (0.198)	0.226
Physical Health	-0.016 (0.005)	**0.005**	-0.009 (0.008)	0.231	-0.021 (0.008)	**0.008**
Mental Health	-0.515 (0.462)	0.265	-0.302 (0.573)	0.599	-0.918 (0.753)	0.223

Table S2. Multivariate Regression Analysis: Clinical and SCL-90 Factors for VAS Back Pain and SF-36 Outcomes, Comparing Interspinous Devices and Pedicle Screw Stabilizations. BMI (Body Mass Index); DMII (Diabetes Mellitus II), SCL-90 (Symptom Checklist-90), GSI (Global Severity Index); PST (Positive Symptom Total); PSDI (Positive Symptom Distress Index); CSI (Current Symptom Index); SF-36 (Short Form 36); SD (Standard Deviation).

Vas back pain	Overall	P-values	Interspinous device	P-values	Screws and Rods	P-values
			Estimate (sd)		Estimate(sd)	
Age	0.011 (0.005)	0.033	0.012 (0.008)	0.144	0.012 (0.009)	0.149
BMI	-0.019 (0.012)	0.110	-0.027 (0.017)	0.108	-0.013 (0.018)	0.452
DM.II	0.023 (0.167)	0.886	0.164 (0.233)	0.480	-0.041 (0.245)	0.864
Fibromyalgia	0.150 (0.381)	0.692	-0.494 (0.536)	0.357	0.735 (0.546)	0.179
Others orthopedic diseases	0.140 (0.139)	0.312	0.318 (0.186)	0.088	0.009 (0.212)	0.965
**SCL90**						
Somatization	0.443 (0.197)	**0.025**	0.208 (0.256)	0.417	0.714 (0.305)	**0.020**
Obsessiveness	0.090 (0.214)	0.674	-0.090 (0.293)	0.757	0.311 (0.319)	0.331
Sensitivity	0.263 (0.225)	0.241	0.290 (0.291)	0.320	0.222 (0.351)	0.527
Depression	0.189 (0.231)	0.414	-0.117 (0.301)	0.696	0.516 (0.359)	0.152
Hostility	-0.204 (0.204)	0.319	-0.244 (0.283)	0.389	-0.235 (0.298)	0.430
Fobic Anxiety	0.341 (0.250)	0.173	-0.268 (0.317)	0.399	1.068 (0.397)	**0.007**
Paranoid Ideation	0.315 (0.163)	0.054	-0.001 (0.204)	0.992	0.733 (0.268)	**0.006**
Psychoticism	-0.491 (0.288)	0.089	-0.246 (0.380)	0.517	-0.648 (0.451)	0.151
GSI	0.004 (0.012)	0.726	0.0006 (0.013)	0.963	0.026 (0.040)	0.515
PST	0.0002 (0.008)	0.973	0.0001 (0.010)	0.985	0.002 (0.013)	0.877
PSDI	0.079 (0.115)	0.489	0.094 (0.149)	0.527	0.120 (0.179)	0.500
CSI	-0.744 (0.730)	0.308	0.451 (0.924)	0.625	-2.317 (1.179)	0.050
**SF-36**						
Age	0.006 (0.004)	0.176	0.006 (0.007)	0.369	0.005 (0.007)	0.520
BMI	-0.027 (0.011)	**0.014**	-0.038 (0.015)	0.0106	-0.015 (0.016)	0.348
DM.II	-0.044 (0.149)	0.763	0.137 (0.201)	0.492	-0.189 (0.224)	0.400
Fibromyalgia	0.235 (0.343)	0.492	-0.054 (0.469)	0.908	0.522 (0.500)	0.297
Others orthopedic diseases	0.186 (0.123)	0.131	0.399 (0.159)	0.012	0.011 (0.192)	0.954
Role limitation due to physical health	0.010 (0.002)	**0.0001**	0.003 (0.003)	0.339	0.013 (0.003)	**0.0003**
Role limitation due to emotional problems	0.090 (0.074)	0.224	0.061 (0.092)	0.505	0.152 (0.121)	0.209
Energie fatigue	0.125 (0.099)	0.206	0.087 (0.122)	0.472	0.205 (0.161)	0.205

Emotional well being	0.153 (0.124)	0.219	0.098 (0.154)	0.525	0.257 (0.202)	0.202
Social Functioning	0.059 (0.049)	0.235	0.038 (0.061)	0.533	0.099 (0.080)	0.218
Pain	-0.026 (0.003)	**0.0001**	-0.033 (0.004)	**0.0001**	-0.021 (0.005)	**0.0001**
General Health	0.154 (0.124)	0.216	0.102 (0.153)	0.504	0.258 (0.202)	0.203
Physical Health	-0.014 (0.005)	**0.014**	-0.003 (0.008)	0.696	-0.021 (0.008)	**0.010**
Mental Health	-0.595 (0.473)	0.209	-0.399 (0.584)	0.494	-0.985 (0.768)	0.200

Table S3. Multivariate Regression Analysis: Clinical and SCL-90 Factors for EQ-5D and SF-36 Outcomes, Comparing Interspinous Devices and Pedicle Screw Stabilizations. EQ-5D (Euro Quality of life 5 Dimension), BMI (Body Mass Index); DMII (Diabetes Mellitus II), SCL-90 (Symptom Checklist-90), GSI (Global Severity Index); PST (Positive Symptom Total); PSDI (Positive Symptom Distress Index); CSI (Current Symptom Index); SF-36 (Short Form 36); SD (Standard Deviation).

EQ-5D	Overall	P-values	Interspinous device	P-values	Screws and Rods	P-values
			Estimate (sd)		Estimate(sd)	
Age	-0.001 (0.0007)	0.052	-0.002 (0.001)	0.061	-0.001 (0.001)	0.173
BMI	-0.0002 (0.001)	0.868	0.0001 (0.002)	0.681	-0.001 (0.002)	0.507
DM.II	0.011 (0.022)	0.607	0.021(0.032)	0.448	0.011 (0.032)	0.711
Fibromyalgia	-0.049 (0.050)	0.325	-0.021(0.007)	0.768	-0.098 (0.071)	0.172
Others orthopedic diseases	0.012 (0.018)	0.506	-0.003 (0.024)	0.872	0.023 (0.027)	0.400
**SCL90**						
Somatization	-0.013 (0.026)	**0.597**	-0.012 (0.033)	0.724	-0.012 (0.040)	**0.757**
Obsessiveness	0.037 (0.028)	0.183	0.071 (0.038)	0.063	0.006 (0.041)	0.875
Sensitivity	0.020 (0.029)	0.495	0.065 (0.038)	0.086	-0.033 (0.046)	0.469
Depression	-0.026 (0.031)	0.394	0.004 (0.003)	0.913	-0.052 (0.047)	0.269
Hostility	-0.033 (0.027)	0.221	-0.002 (0.003)	0.955	-0.037 (0.039)	0.340
Fobic Anxiety	0.008 (0.033)	0.796	0.051 (0.041)	0.222	-0.035 (0.052)	**0.493**
Paranoid Ideation	-0.011 (0.021)	0.586	-0.013 (0.021)	0.624	-0.016 (0.035)	**0.647**
Psychoticism	0.099 (0.038)	0.009	0.096 (0.050)	0.055	0.079 (0.059)	0.178
GSI	0.005 (0.001)	0.001	0.0061 (0.001)	0.0005	0.003 (0.005)	0.525
PST	-0.0005 (0.001)	0.637	-0.00005 (0.0014)	0.974	-0.001 (0.001)	0.479
PSDI	-0.057 (0.015)	0.0001	-0.089 (0.019)	0.0001	-0.031 (0.023)	0.184
CSI	-0.145 (0.096)	0.131	-0.293 (0.121)	0.016	0.020 (0.154)	0.894
**SF-36**						
Age	-0.0005 (0.0006)	0.361	-0.0006 (0.001)	0.548	-0.001 (0.0009)	0.306
BMI	-0.0005 (0.001)	**0.736**	0.002 (0.002)	0.182	-0.003 (0.002)	0.141
DM.II	0.018 (0.019)	0.367	0.015 (0.028)	0.579	0.027 (0.028)	0.333
Fibromyalgia	-0.043 (0.046)	0.345	-0.031 (0.066)	0.632	-0.068 (0.063)	0.286
Others orthopedic diseases	0.002 (0.016)	0.861	-0.027 (0.022)	0.225	0.020 (0.024)	0.397
Role limitation due to physical health	0.0004 (0.0003)	**0.216**	0.001 (0.0005)	0.021	0.0001 (0.0004)	**0.804**

Role limitation due to emotional problems	-0.023 (0.010)	0.017	-0.032 (0.012)	0.012	-0.017 (0.015)	0.270
Energie fatigue	-0.031 (0.013)	0.020	-0.042 (0.017)	0.013	-0.021 (0.020)	0.300
Emotional well being	-0.039 (0.016)	0.018	-0.053 (0.021)	0.013	-0.027 (0.025)	0.286
Social Functioning	-0.015 (0.007)	0.022	-0.020 (0.008)	0.018	-0.011 (0.010)	0.279
Pain	0.001 (0.0004)	**0.006**	0.001 (0.0006)	**0.006**	0.001 (0.0006)	**0.143**
General Health	-0.040 (0.016)	0.015	-0.055 (0.021)	0.010	-0.028 (0.025)	0.278
Physical Health	0.003 (0.0007)	**0.003**	0.003 (0.001)	0.003	0.002 (0.001)	**0.007**
Mental Health	0.151 (0.063)	0.017	0.205 (0.082)	0.013	0.107 (0.098)	0.271
